# The hippocampus as a generative model

**DOI:** 10.1098/rstb.2025.0247

**Published:** 2026-07-09

**Authors:** Sumedha Nalluru, Leonie Glitz, Xenia Przygodda, Valentina Mancini, Helen C. Barron

**Affiliations:** 1 Medical Research Council Centre of Research Excellence in Restorative Neural Dynamics, Nuffield Department for Clinical Neurosciences, University of Oxford, Oxford, United Kingdom; 2 Oxford Centre for Integrative Neuroimaging, University of Oxford, FMRIB, John Radcliffe Hospital, Oxford, United Kingdom; 3 Department of Experimental Psychology, University of Oxford, Oxford, United Kingdom; 4 Department of Psychiatry, University of Oxford, Oxford, United Kingdom

## Abstract

A generative model can be defined as a model of the latent causes of sensory input that can be used to generate new data samples. By examining empirical evidence and computational theory, we propose that the hippocampus can be characterised as a generative model. The hippocampus is a brain region important for memory. Recordings of neural activity from hippocampus have led to the view that the hippocampus represents a cognitive map by abstracting a low-dimensional representation of the external world. We extend this view to suggest the hippocampus represents the latent, unobserved causes of sensory data, by virtue of the position of the hippocampus within the deep cortical hierarchy. These representations of unobserved latent causes endow the hippocampus with capacity to generate new data samples that allow exploration of future hypotheticals and provide an internally generated training signal back to the generative model. We explore how perturbations to the hippocampal generative model may explain core symptoms of neuropsychiatric disorders, such as those observed in psychosis. Together, this perspective provides a unified account of hippocampal function that explains how computations performed by the hippocampus support higher-order cognition and adaptive behaviour.

## What is a generative model?

Generative models have long been used to explain complex behaviours [1–3]. However, the neural circuit implementation is only beginning to be fully realised via an ongoing dialogue between artificial intelligence (AI) and neuroscience. Here, we propose that the hippocampus, a brain region important for memory, may be conceptualised as representing the apex of a generative model that is represented by the cortical hierarchy. Before examining evidence to support the view, we first provide a conceptual and computational definition of a generative model.

To define what we mean by a generative model, first we consider how the brain receives sensory input and uses this input to guide perception and behaviour. For example, a child may see a banana from different locations. While the exact visual input is not memorised, each time the child sees the banana, visual input provides training data to extract and learn a hidden (or ‘latent’) state (in this case, ‘banana’). This latent state is unchanging despite variation in the sensory input, illustrating how perception itself requires inference and is inherently constructive. Beyond perception, more abstract latent states can be inferred. For example, a child may have to leave the house to infer that it is raining (wet pavement, puddles, etc.), yet over time, a diverse set of sensory inputs (raindrops on the window, pitter patter sound outside) provide training data to extract and learn the hidden (or ‘latent’) state (in this case, ‘rainy day’). By representing latent states, the brain is endowed with capacity to generate and imagine entirely new examples (e.g., a new banana, rain tomorrow). These examples illustrate the core function of a generative model: past experiences provide training data to learn and later infer hidden (or ‘latent’) rules that can be used to generate new outputs.

From a computational perspective, a generative model can be defined as a model of the causes and states that generate observed sensory data. These causes and states are ***not directly observed*** but are instead ***inferred***, and therefore described as ***‘latent’***, ‘hidden’ or ‘unobserved’.

We refer to the process of inferring latent causes from sensory data as ***perceptual inference***. Perceptual inference can be mathematically formalised using a ***Bayesian framework*** [4,5]. Given sensory data *X*, the probability of latent cause *Z* is described as the ***posterior*** and formalised as: p(Z|X). The posterior is estimated by combining the ***likelihood*** of observing the sensory data (p(X|Z)) with ***prior*** beliefs (p(Z)) that are held about the environment.

In addition to perceptual inference, the generative model can be used to ***generate new data samples***, by calculating the probability of sensory data *X* given the model’s representation of the latent cause *Z* (i.e. p(X|Z)). Thus, latent state representations are used to reconstruct or simulate sensory experience. Generating new data samples can serve several purposes. First, new data can be used to ***predict future observations***. These predictions can be defined as the model’s internally generated expectations of sensory data, given the assumptions or beliefs about the world represented by the generative model. By predicting future observations, the generative model may explain incoming sensory data before it arrives, to minimise surprise, or even represent future hypotheticals to guide planning and inform decision-making. When these predictions extend beyond past experience, the generative model can be used to ***infer new relationships*** that have not been directly observed, thus enabling adaptive and flexible behaviour.

Second, new data samples can be used to provide ***training data*** to neural circuits, to ***drive learning*** in the absence of sensory input. This training signal may manifest as an internally generated prediction error signal, using the mismatch between: *(a)* latent state representations used to simulate sensory data in low levels of the hierarchy; and *(b) inferred* latent state representations, where the simulated sensory data is used as an input for perceptual inference. Thus, in the absence of sensory data, the resulting prediction error signal can be used to update neural circuits, including the parameters of the generative model itself.

From a computational perspective, the Helmholtz machine [1] provides an intuitive example of how a generative model may be implemented in neural architecture. In brief, the Helmholtz machine is a hierarchical model that uses a recognition model to infer the latent causes of the observed sensory data. The recognition model allows for the inversion of the internal world model, to enable inference of latent causes from sensory data. This recognition model is then coupled with a generative model capable of generating sensory data from latent causes represented by the internal world model.

In the Helmholtz machine, learning is achieved via a “wake-sleep” algorithm [6] that separates opportunities for learning. During the wake phase, sensory data from the world drives the lowest layers and activity propagates up to higher layers, where the underlying cause is inferred using a feedforward approximation of the posterior. During the sleep phase, the generative model is used to sample latent variables (or priors), thus generating example data, which is first represented by the top-layers before activity propagates to successively lower layers. Since the generative model represents the true underlying cause for the generated data (for example, ‘banana’), the target value of the hidden units is known, providing a means to train synaptic weights in lower layers of the hierarchy. In other words, the sleep phase uses self-generated training data to train the recognition model to perform rapid feedforward approximation of the posterior. Notably, the generative model allows the recognition model to invert the generative model for inputs distributed according to probability distributions in the generative model rather than distributions in real data. In this manner, the generative model calibrates perceptual inference. The Helmholtz machine is arguably the conceptual precursor for incorporating generative capacity in modern AI, including Variational Autoencoders (VAEs), where VAEs learn how to represent a set of probabilistic latent variables that can be sampled to generate new data. VAEs have been used to provide elegant computational accounts of the hippocampus as a generative model [7,8].

Here we explore how the hippocampus, a brain region important for memory, can be characterised as a generative model [7–18]. Our perspective aims to provide a unique synthesis of empirical data and computational theory. For example, we explore the distinct anatomical position of the hippocampus, which sits at an apex in the cortical hierarchy and receives converging inputs from disparate neural circuits. We suggest, therefore, that the hippocampus is uniquely positioned to execute the dual aspects of a generative model. Namely, during ‘online’ behaviour the hippocampus can contribute to *perceptual inference*, while in ‘offline’ periods of rest or sleep, the hippocampus has unique capacity to intrinsically generate spiking sequences that resemble *self-generated training data*. The synergy of these two aspects of a generative model may be unique to the hippocampus, yet the underlying computations operate in concert with other circuits. For example, perceptual inference is implemented via message passing up the cortical hierarchy, while spiking sequences that are intrinsically generated in hippocampus can propagate down the cortical hierarchy, to even predict activity in primary sensory cortices [19–21]. We highlight how these mechanisms interact with neuromodulatory systems, such as the dopaminergic system. Moreover, we explore how higher order brain regions, such as entorhinal cortex and prefrontal cortex, may provide structure both to hippocampal representations of latent causes, and spiking sequences generated within the hippocampus. Taken together, our perspective provides a comprehensive characterisation of the hippocampus as a generative model which formulates a natural extension to the more traditional view that the hippocampus represents a cognitive map [22–27].

Overall, by conceptualising the hippocampus as a generative model, we describe how the hippocampus contributes to broad aspects of higher-order cognition that extend beyond memory per se, to include state representation, decision-making, inference, imagination, planning, continual learning, and the extraction of statistical regularities. Further, by examining how temporal sequences within the hippocampus provide a generative readout in time, we account for representations of latent *temporal* structure in hippocampus, both during abstract tasks [28] and in response to rich narratives [29]. Finally, we discuss how perturbations to the hippocampus may disturb the capacity of the generative model to support adaptive behaviour. Using psychosis as a case study, we suggest that perturbations to the hippocampal generative model can account for core clinical symptoms in psychosis, including hallucinations and delusions.

## From cognitive map to generative model

The traditional view of the hippocampus is that it creates a cognitive map that can be used to navigate both physical and more abstract spaces. The term cognitive map was first introduced by Tolman to explain behaviour in rodents. Namely, after being exposed to a spatial maze, rats were able to infer shortcuts to obtain rewards [22] and infer new routes when old routes were blocked [23]. To account for these behaviours, Tolman suggested that the mammalian brain represents a rich internal model of the world or cognitive map that provides a means to predict the consequence of actions, including those that involve novel trajectories.

The discovery of ‘place cells’ in the rodent hippocampus provided the first evidence for a neural implementation of the cognitive map. Place cells constitute pyramidal cells in the hippocampus and show elegant tuning to specific spatial locations [24]. Together with a rich menagerie of other spatial codes reported in the hippocampus and neighbouring brain regions, place cells provide a model of *where* an agent is in absolute space. The model serves a memory system by representing locations in an organism’s environment, their spatial relations and the existence of specific objects in specific places. This allows an agent to locate itself and navigate within a familiar environment without reference to any specific sensory input. O’Keefe and Nadel described this model as a “cognitive map”: a neural system which generates a model of absolute space [24]. Equivalent neural codes can be observed for non-spatial relationships [25,26], suggesting that the hippocampal cognitive map represents relationships that not only help us navigate the spatial environment but also more abstract relationships and knowledge [27].

Here, by exploring the broader anatomical position and function of the hippocampus, we seek to extend this view to suggest the hippocampus can be conceptualised as a ***generative model***. As outlined above, the first key function of a generative model is to perform perceptual inference by inferring and representing the latent causes of sensory data which are not directly observed [30]. Below we examine theoretical and empirical evidence to suggest the hippocampus is ideally positioned to support perceptual inference on highly processed sensory input [7,16,18]. Within this view, the hippocampus does not merely represent a cognitive map or internal model (here after used interchangeably) built from past experience (or memory). Rather, the hippocampus represents the inversion (or approximate inversion) of the internal model to map observed sensory data to the latent (unobserved) causes or states (i.e., ***perceptual inference***). In other words, the hippocampus represents the posterior as a latent cause that draws both from past experience *and* incoming sensory data. Within this framework, place cells may be interpreted as representing the unobserved location in the cognitive map that is inferred from sensory input, namely the outcome of latent state inference.

However, often the inferred state is more abstract than a spatial location. For example, the latent cause for a particular sensory experience may resemble a particular trajectory through an internal model [8] which may be highly abstract relative to our perceived structure of the world. It is difficult to conceptualise how such highly abstract latent causes should be represented. To a human observer of neural data, activity may be assumed to map onto fundamental axes of perceived structure, such as space and time. However, in the brain the relevant metrics for an internal model are those that can be utilized by downstream neural observers [31]. Therefore, when an agent traverses a linear track, the latent causes for the sensory input may appear to be represented as locations in space (via ‘place cells’) and can be plotted as a trajectory on a spatial map. When an agent traverses a more abstract experience, such as holding a conversation with a colleague, latent causes for the sensory input may be represented in a manner that is more difficult to interpret, simply because the low dimensional embedding of the representation does not readily map onto familiar dimensions of space and time.

The second key function of a generative model is to ***generate new data samples***, by constructing data samples from the representation of latent causes. As discussed below, the hippocampus has unique capacity to intrinsically generate new data samples (i.e., ‘replay’). While a cognitive map can be used to draw new data samples, generative models provide greater explanatory power by naturally accounting for the computational machinery needed to intrinsically generate new data. Thus, we explore both theoretical and empirical evidence to support the view that the hippocampus both represents latent causes and has capacity to generate new data samples. In doing so, we characterise the hippocampus as a generative model, which naturally extends and formalises the notion of a cognitive map within a broader computational framework.

## The hippocampus represents and generates new latent causes

In this section we define how the hippocampus contributes to inferring the latent and unobserved causes of sensory data (“***perceptual inference***”). By examining empirical data and theoretical frameworks, we propose that latent state inference is computed across the cortical hierarchy, where the unique contribution of the hippocampus and related circuits is to create and utilise representations of ***new latent causes***. Notably, perceptual inference is not a feature of several popular computational accounts of hippocampal function, such as the successor representation [32,33], which otherwise explain a number of properties of place cell and grid cell activity.

Although empirical evidence for perceptual inference predominantly derives from visual cortices [34], this mathematical framework can be applied to the entire cortical hierarchy. Therefore, at each level of the cortical hierarchy, descending predictions (or priors) that originate in high levels of the cortical hierarchy are integrated with ascending sensory inputs to infer the latent cause by estimating a ***posterior***. Over time, as dynamic message passing continues to ascend and descend the cortical hierarchy, the representation of a latent cause in lower levels of the cortical hierarchy may become increasingly abstract.

While it remains experimentally difficult to assess dynamic message passing across the cortical hierarchy, both computational modelling and empirical evidence suggest representations of latent states can be found in higher-order brain regions, including the prefrontal cortex and orbitofrontal cortex, as discussed elsewhere [37–39]. Here, we focus on the contribution of hippocampus to latent state inference. First, causal manipulations in mice demonstrate that the hippocampus is necessary for inferring latent context [40]. Second, the phenomenon of hippocampal remapping suggests the hippocampus represents latent causes. Hippocampal remapping can be defined as the change in preferred spatial tuning or firing intensity of place cells between different environments. Therefore, at the population level, remapping involves a change in the map that tiles the environment. This change in the map can involve ***global remapping*** where the preferred spatial tuning of hippocampal place cells changes, or ***rate remapping***, where the preferred spatial tuning remains the same, but the firing rate changes [41].

The occurrence of rate versus global remapping appears to depend on both the nature of the environmental change, and whether the animal infers that it is in a new context (latent cause). For example, global remapping is observed in response to substantial changes in sensory cues [42,43] that are sufficient to suggest a change in context represented as a new latent cause. In line with theoretical predictions [8], global remapping is not random but rather involves structured realignment of population activity [44,45]. This structured realignment may be determined by latent structural knowledge represented by grid cells in entorhinal cortex [8]. Global remapping in the hippocampal population code can be interpreted as representing a different latent state.

**Figure 1:**
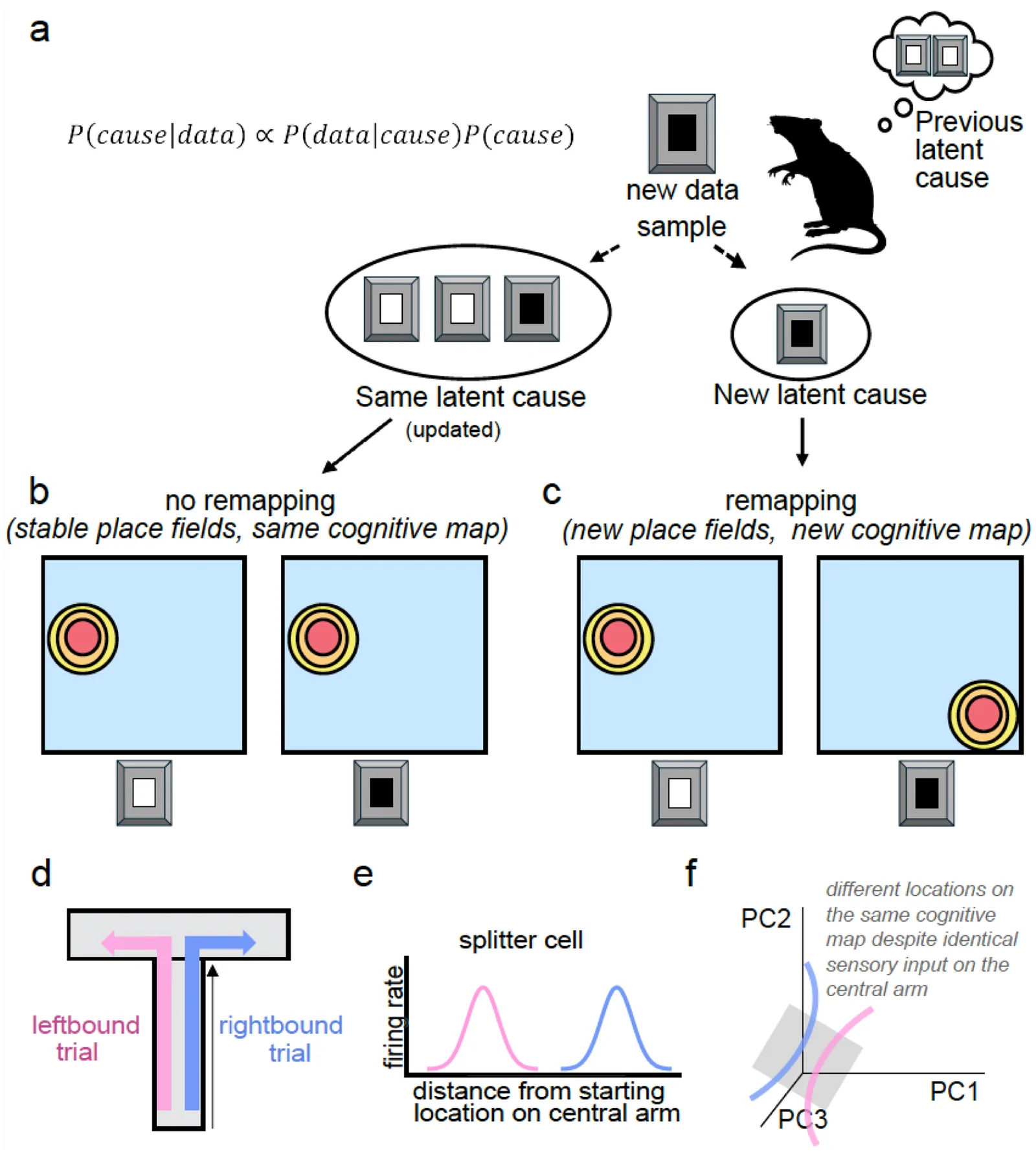
The hippocampus represents the posterior of latent state inference. **(a-c)** In conjunction with other brain regions, the *(a)* probability of the current latent cause given incoming data is computed (illustration adapted from [35]). If the incoming data is *(b)* attributed to the same latent cause, the hippocampal place cell code remains the same. If the incoming data is *(c)* attributed to a different latent cause, hippocampal remapping occurs to represent the new latent cause, which resembles a new cognitive map. **(d)** Example of a T-maze, where animals perform an alternation task (run to left arm, right arm, left arm) to obtain reward. **(e)** Schematic showing an example splitter cell in the hippocampus which represents different locations on the central arm of the T-maze depending on whether the upcoming trial is a left- or right-bound trial. **(f)** Schematic showing that when projected into a low-dimensional space, the activity of splitter cells reflects different locations within a cognitive map which may reflect a representation of two distinct latent causes, despite identical sensory input; *(e)* and *(f)* adapted from [36].

Rate remapping, on the other hand, can be observed in response to more subtle changes in sensory cues. For example, Leutgeb et al., [41] recorded spiking activity in the hippocampus while rats were placed within a square environment with a specific wall colour. The colour of the walls was varied (either black or white), while leaving all other local and global cues in the environment intact. When the wall colour changed, the firing rate of pyramidal cells changed but their preferred spatial tuning remained the same (rate remapping) (Figure 1a-c). This rate rather than global remapping in the hippocampal population code can be interpreted as maintaining the same latent cause (i.e. same environment) despite changes in sensory cues.

In addition to hippocampal remapping, so called ‘splitter cells’ provide the hallmark characteristic of a neural representation for latent causes (Figure 1d-f). While the traditional definition of a ‘place cell’ is a neuron that fires at a particular spatial location, ‘splitter cells’ transcend this definition by showing firing responses that are modulated by an animal’s recent choice history or upcoming goal [36,46,47]. For example, as an animal traverses the vertical arm of a continuous ‘T’ maze, the firing of a splitter cell will depend upon both the spatial location and whether the animal intends to take the left or right turn at the top of the vertical arm [48].Therefore, the information represented by these cells extends beyond incoming sensory and motor input to include more abstract information about the recent past and/or upcoming choice. When the activity of neuronal ensembles is plotted in low dimensional space, the neural activity appears to be ‘pulled apart’ or split by a hidden variable, despite the animal traversing the same physical location [49]. Therefore, activity in splitter cells does not necessarily correspond to observable states but instead reflects the agent’s beliefs about the underlying structure of the environment, given incoming sensory data [36]. Moreover, the activity of splitter cells can generalise across tasks with identical structure [49,50], providing further evidence to suggest that splitter cells may represent latent causes.

The phenomena of hippocampal remapping and splitter cells both provide evidence to support the view that the hippocampus represents unobserved latent causes of sensory input. These representations of latent causes may be inherited from another brain region or constructed anew in hippocampus itself. By examining fear conditioning paradigms, we suggest that the hippocampus can play an active role in constructing representations of *new* latent causes. Here, an animal first undergoes fear conditioning (‘conditioning phase’, sound paired with a shock), before undergoing extinction (‘extinction phase’, sound without shock) until the animal no longer freezes in response to the sound. If the animal is later returned to the shock context, ‘renewal’ behaviour is typically observed, where the animal shows a fear response (freezing to sound without shock). Notably, renewal behaviour is observed when the extinction phase is introduced rapidly. The expression of renewal behaviour suggests that two separate latent causes (or posterior distributions) are used to explain the conditioning and extinction phase of the task. A mature and intact hippocampus appears necessary to infer new latent causes in response to rapid extinction, as both adult rats with hippocampal lesions and young rats with an intact hippocampus display a lack of renewal behaviour following fear conditioning with rapid extinction [51–53]. More nuanced reports of the effect of hippocampal lesion on renewal-like behaviour suggest that pronounced deficits are observed when both the extinction and conditioning occur within the same context [54]. This suggests that the hippocampus contributes to constructing latent state representations when there is sufficient ambiguity as to whether a new latent cause is appropriate, while alternative brain regions, such as prefrontal cortex and amygdala, may have capacity to compensate in certain paradigms [55].

In contrast to rapid extinction protocols, if extinction is introduced gradually, renewal is less pronounced. For example, following a gradual fear extinction paradigm in rats, Gershman et al. found no evidence for recovery of fear [35]. This suggests that gradual extinction promotes representation of a single latent cause for both the conditioning and extinction phases of the task. To provide a mechanistic account, one possibility is that in a gradual extinction condition, the total prediction error across time is not sufficient to trigger inference of a new latent state. Rather, the latent state generated during the conditioning phase is slowly updated to gradually increase the likelihood of an extinction trial.

Dopaminergic modulation may play a key role in signalling the need for hippocampus to infer a new latent cause. When incoming sensory data is unexpected given prior beliefs, the resulting mismatch in expectation generates a dopaminergic prediction error signal. Redish et al. have proposed a theoretical model in which new latent causes are inferred from sensory data when a sufficiently large negative prediction error is encoded by dopamine, a process described as ‘state-splitting’ [10]. Here, acquisition of new learning and extinction are modelled as two interacting learning processes, which are driven by positive and negative reward prediction error signals, respectively. However, alternative theories suggest the magnitude, rather than the sign, of the prediction error determines ‘state splitting’, where a small prediction error drives incremental learning, while a large prediction error generates a new state [13].

While empirical evidence is needed to arbitrate between these alternatives, the known anatomy supports the view that dopaminergic projections target hippocampus, providing a potential pathway to update and/or construct latent state representations. For example, dopaminergic fibres from the ventral tegmental area (VTA) preferentially innervate the output regions of the hippocampus (CA1 and subiculum) and causal manipulations demonstrate that these projections contribute to hippocampal-dependent memory performance [56]. In addition, projections from locus coeruleus (LC) to hippocampus are profuse and co-release dopamine with noradrenaline [57,58]. These LC projections to hippocampus signal a dopamine-dependent novelty effect which can mediate hippocampal learning [59]. VTA and LC dopaminergic innervation of hippocampus may therefore provide prediction error-like signals that drive inference of new latent states, with VTA conveying a signed reward-prediction error signal and LC conveying a novelty signal. Conceptualising the hippocampus as a generative model therefore leads to the prediction that dopaminergic innervation of the hippocampus is necessary to infer new latent states.

Theoretical and empirical work suggests that generative capacity within the hippocampus is also influenced by interactions between hippocampus and other higher-order brain regions, such as the medial entorhinal cortex (mEC). More specifically, mEC is attributed with constraining the relevant set of possible latent causes (or posteriors) represented by hippocampus. Firing activity in pyramidal neurons in the mEC tiles both physical and abstract spaces using a grid-like lattice [25,60–62]. Mathematically, this grid-like code can be formulated as the low dimensional embedding of activity in hippocampal pyramidal cells. Specifically, for place cells that represent location within a 2D environment, grid-cell like firing can be obtained from the eigenvectors of the covariance of the 2D place cells, using the output of a Principal Component Analysis (PCA) with non-negative weights [33,63]. The eigen code in mEC therefore provides an efficient means to capture the variance or structure of activity in the hippocampal population [27]. Neuroimaging studies in humans and computational models both provide evidence to suggest that the mEC provides an explicit representation of structure [8,18,28,33,64,65]. An interesting prediction that arises from this interaction between mEC and hippocampus is that the structural constraint imposed by mEC invokes non-random hippocampal remapping [8,15].

Insight into the mechanism by which mEC structural codes are generated and used can be gained by examining anatomical connections and causal relationships between the mEC and hippocampus. The CA1 region, which serves as a primary output region of the hippocampus, receives inputs from two alternative pathways: the trisynaptic path (via EC → Dentate Gyrus → CA3 → CA1) and the monosynaptic path (via EC → CA1). When considering the monosynaptic path, deep layers of EC (layer V/VI) receive direct projections from CA1, while superficial layers of EC (layer III) send direct projections back to CA1. Grid cells are primarily found in the superficial layers of mEC, providing opportunity to constrain CA1 activity using a structural code. In the temporal domain, this structural constraint on CA1 is reflected in theta phase precession. Bilateral inhibition of mEC disrupts theta in CA1 [66] and mEC lesions disrupt CA1 phase precession even during periods of stable spatial firing [67]. Together these observations suggest that mEC can both constrain the possible set of latent causes represented by CA1 and influence the precise temporal structure of these CA1 representations during active behaviour.

In addition to mEC, other entorhinal subregions may play an important role. For example, the lateral entorhinal cortex (lEC) projects to dopaminergic regions (VTA and substantial nigra,[68]) and, in turn, receives strong dopaminergic inputs from these regions [69]. Dopaminergic prediction errors across lEC and CA1 may therefore contribute to switches in the preferred set of latent causes. At a physiological level this may manifest as a phase shift in the grid cells (akin to grid cell remapping [70]). The interaction between CA1, lEC and dopaminergic prediction error signals in midbrain may therefore determine both whether a new latent state is represented in hippocampus, and which set of possible latent states can be represented by hippocampus.

## The hippocampus generates new data samples

**Figure 2:**
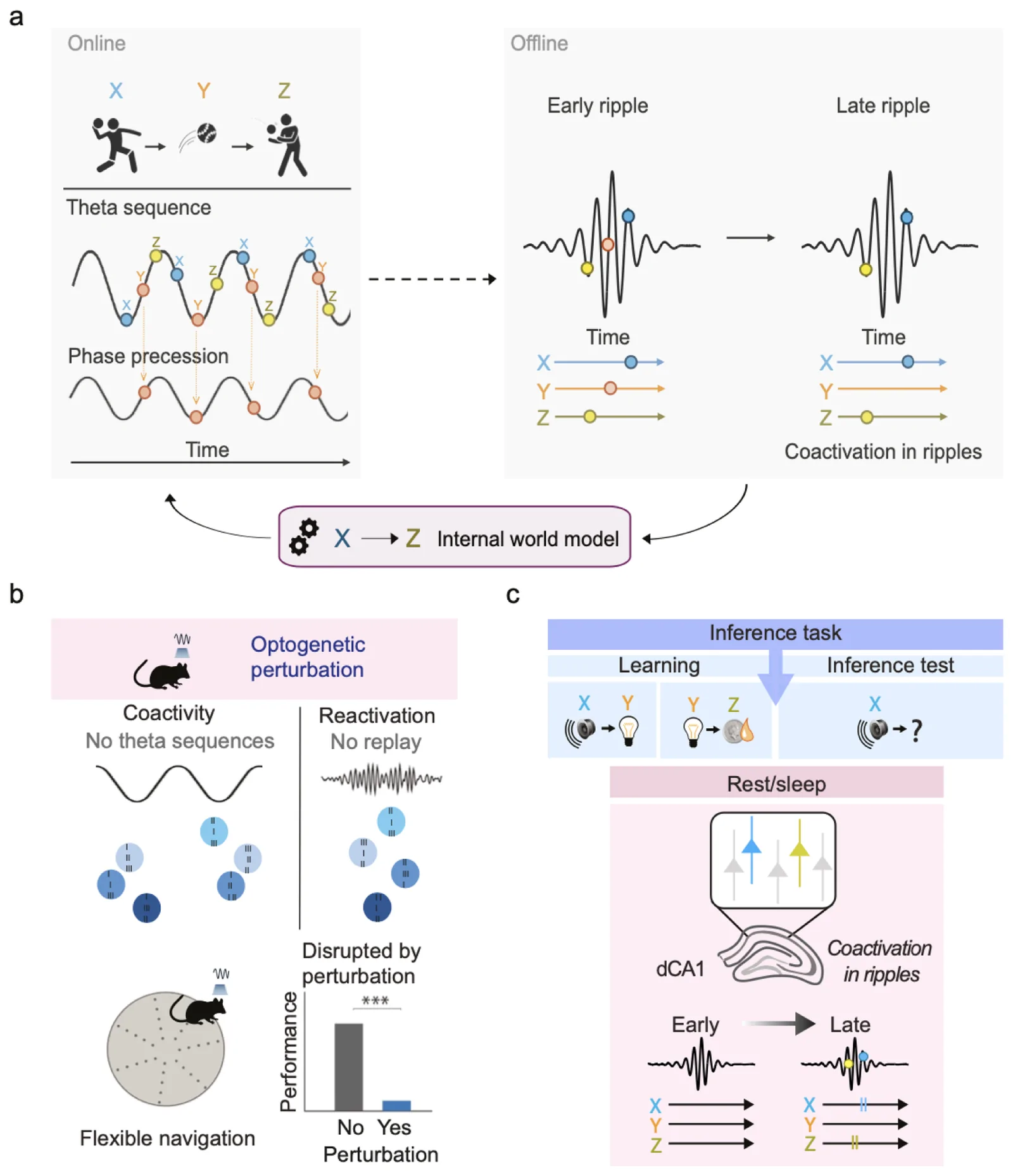
Hippocampal generative sequences in theta and SWRs **(a)** *Left: (Online)* Schematic illustrating how theta phase precession organizes sequential elements within individual theta cycles. Each sequence element (X, Y, Z; indicated by both colour and letter labels) is represented by coloured dots aligned to successive theta phases, producing a theta sweep from past (X) through present (Y) to future (Z). In this example, *X* corresponds to seeing a ball being thrown, *Y* to tracking the ball’s trajectory, and *Z* to seeing the ball get caught. *Right: (Offline)* Schematic showing generative replay during hippocampal SWRs in periods of rest and sleep. Early SWRs replay sequences of events (*X, Y, Z*). Over time, in late ripples, coactivation of *X* and *Z* is observed, without intermediary *Y*. Generative sequences across theta and SWRs may influence one another, such that plasticity from theta phase precession constrains the content of generative replay, while generative replay may provide a SSL signal to update the generative model. In turn, this influences theta phase precession upon re-exposure. **(b)** Figure reproduced with permission from Liu et al., [128]. They disturb the temporal order of theta sequences in rats using an optogenetic manipulation during ongoing spatial navigation. This manipulation disturbs the precise order SWR firing sequences in subsequent periods of rest, suggesting that the content of theta sequences influences the content of generative replay. **(c)** Barron et al., [113] show in mice that shortcuts in the generative world model emerge over the course of hippocampal SWRs, with late SWRs skipping directly learned associations to instead represent inferred relationship.

In addition to perceptual inference, generative models have capacity to ***generate new data samples*** (Figure 2). These new data samples can be directly (re)constructed from the representation of latent causes. The capacity to generate new data samples provides an efficient computational solution to many aspects of higher-order cognition, such as memory consolidation, continual learning, the prediction of future observations and inference of new relationships. Here we examine firing sequences reported in the hippocampus that reflect this aspect of generative activity.

First, we consider firing sequences that occur in the hippocampus during theta oscillations. During online periods, when animals or humans engage with a task, hippocampal pyramidal neurons fire at systematically earlier phases of the theta cycle (4–12 Hz in rodents, 4–8 Hz in humans) as the animal [71–76], or human [77–80] traverses through the cell’s place field (Figure 2a). This phenomenon is known as ***theta phase precession***. While phase precession was first observed across place cells, the phenomenon has since been reported for more abstract, non-spatial sequences [26]. At the population level, coordinated phase precession across ensembles can give rise to theta sequences, which can be described as a ***theta sweep***: a temporal ordering of neural activity that spans past, present, and potential future locations within a single theta cycle. On a linear track and in a T-maze environment forward directed theta sweeps are observed [76,81,82], while in an open field, sweeps are left-right alternating [83]. We note that phase precession can occur in individual neurons in the absence of coherent population-level theta, reflecting an intrinsic property of place cells [72]. Therefore, while phase precession can be observed in individual neurons without prior experience, ensemble-level theta sequences develop rapidly with exposure to a new spatial maze [84], becoming stronger with experience [85]. Thus, theta sweeps, rather than phase precession intrinsic to individual neurons, may reflect rapid exploration of possible future trajectories to predict upcoming behaviour over a short-time horizon [81,86,87], although a reliable relationship between theta sweeps and behaviour is not always observed [70]. In addition to representing the immediate future, theta sweeps are also reported to represent counterfactual trajectories [82] and distal spatial locations [88], suggesting that reconstructed data samples have the potential to be non-local.

Theta phase precession therefore provides a candidate generative mechanism where late theta phases reflect new data sampled from latent causes. Examining the anatomy of the hippocampus adds nuance to this view. Along the tri-synaptic pathway (via EC → Dentate Gyrus → CA3 → CA1), recurrent connectivity within CA3 has traditionally been considered to support memory recall or pattern completion. Here we suggest that recurrent connectivity within CA3 endows CA1 with capacity to receive internally generated data samples that are reconstructed from an internal world model. By integrating inputs from this tri-synaptic pathway with those of the monosynaptic pathway (via EC → CA1), CA1 has opportunity to compute a prediction error signal between generative activity and incoming sensory data (from EC) [89]. Empirical data supports this view, with hippocampal BOLD signal in humans covarying with the magnitude of prediction errors [90–93].

A second candidate firing pattern for generative hippocampal activity occurs during Sharp-Wave Ripples (SWRs): high-frequency oscillations that dominate the hippocampal local field potential during offline periods of rest and sleep. The consensus view is that the genesis of sharp-wave occurs within the highly recurrent CA3 sub-region of the hippocampus, which is endowed with the high-frequency ripple generated by fast-spiking interneurons in CA1 [94]. During SWRs, firing activity in the hippocampus appears to ‘replay’ past experience on a temporally compressed scale that is concomitant with the induction of synaptic plasticity [88,95]. Selective disruption of hippocampal SWRs impairs learning and memory, demonstrating a causal role between SWR replay and memory consolidation [96–98]. In AI, the computational advantages of replay can be demonstrated in a continual learning setting. Continual learning is the ability to demonstrate new learning, without rapid forgetting of what was learned before. Therefore, when a computational model has to learn multiple tasks, replay of previous experience (‘experience replay’) [99] can help protect the model from catastrophic forgetting, such that learning a new task doesn’t necessarily interfere with performance on an older task. In the brain, firing sequences during SWRs may therefore be conceptualised as providing a training signal that consolidates new learning while protecting against memory interference. Indeed, while SWRs are intrinsically generated within the hippocampus, the SWR and sequences generated therein can propagate across the cortical hierarchy, to potentially drive consolidation in distributed circuits that extend beyond the hippocampus [98,100]. When propagating across circuits, the temporal structure of the firing sequences must be preserved despite varying intrinsic timescales, to construct and propagate rich narrative structure [29]. Taken together, both prediction error signals and replay that are initially local to the hippocampus may reach all the way out to sensory cortex, providing an internally generated ***training signal*** to embed a hierarchical generative model across cortex.

However, in addition to merely replaying past experience, evidence suggests replay is generative, with spiking activity during SWRs representing sequences that extend *beyond* direct experience. One possibility is that this ***generative replay*** provides a ***signature of planning***. For example, when rats perform goal-directed spatial navigation in an open arena, SWR-related firing sequences predict immediate future behaviour [101]. Moreover, SWR-related firing sequences can depict evaluation of potential options at a decision point [102] and reflect traversals of an aversive path before an animal shows avoidance behaviour [103]. However, when the content of awake SWRs is directly related to both past and future behaviour, replay content is found to be decoupled from subsequent choice [104]. Instead, replay content represents previously rewarded locations and places that had not been visited recently [104]. This suggests that generative replay may not provide a signature of planning for immediate upcoming behaviour. Rather, as explored in numerous computational models and theoretical perspectives [105–109], generative replay may further protect against catastrophic forgetting [110] and reflect adaptive computations that train a generative model to optimise future behaviour, rather than directly dictating immediate choices.

To optimise future behaviour, generative replay can provide virtual exploration of hypothetical trajectories to ***maximise future reward***. Replay has long been considered a mechanism for memory consolidation [98]. Moreover, replay is implicated in computational models, such as the successor representation [111,112], which use replay to rapidly propagate reward information and learn long-range predictive structures, to efficiently assign credit and learn multi-step relationships that extend beyond direct experience. Empirical data support this view. For example, spiking activity during SWRs can represent spatial trajectories that have never been experienced by an animal [103], future event locations [102], spatial trajectories to future goal locations [101] and short-cuts that support inference to a reward [113]. The direction of generative replay further suggests that sequences anchor around real and/or hypothetical rewards. For example, during waking behaviour, replay often proceeds in the forward direction to prospectively represent trajectories toward future goals [101,102], whereas receipt of reward is typically proceeded by reverse replay [113–115]. This reverse replay provides a potential mechanism to backpropagate reward or assign credit to the start of a trajectory [101,114], or even to a non-contiguous cue that logically leads to reward [113] (Figure 2c). This account may be extended to salience attribution to otherwise neutral cues, given evidence to suggest that novel, surprising, or affective memories are preferentially replayed [56,96,116].

Causal manipulations in humans further support the idea that generative replay favours hypothetical trajectories to maximise future reward. These causal manipulations use a technique called Targeted Memory Reactivation (TMR), which involves pairing sensory cues with specific memories during learning, before re-exposing participants to these cues during offline periods of sleep/rest [117–119]. This re-exposure biases firing sequences within hippocampal SWRs towards the cued associations [120], thus providing a tool to causally manipulate the content of replay. The behavioural consequences of TMR vary depending on when and how TMR is delivered. When TMR is applied as a contextual manipulation during awake rest, TMR can bias reactivation towards an entire set of memories without interfering with the endogenous replay sequences. Thus, contextual TMR can be used to enhance generative replay across stimuli or memories associated with the contextual cue. Intriguingly, following this TMR manipulation, participants are subsequently more accurate at inferring the value of an outcome, while no effect is observed for behaviours that draw directly from previous experience [121,122]. This suggests that rather than merely supporting memory consolidation or planning, firing sequences during SWRs can reflect the resampling of fictive sequences from a generative model [18]. These fictive sequences may provide an efficient means to explore ***both real and hypothetical trajectories***.

For generative replay to be adaptive, the generated sequences must be constrained to a set of probable or plausible possibilities. In other words, the rules or grammar defining the structure of generative replay must be non-random. The empirical data discussed above provides some insight into these rules, suggesting that replay is shaped by behavioural relevance, which is influenced by reward and salience [98,100]. Computational modelling provides additional insight into the rules that govern the content of generative replay. Within a reinforcement learning framework, generative replay favours trajectories that update those internal representations (priors) with greatest potential to reduce future uncertainty or maximise reward [105,123]. When considering generative replay and structural learning in concert, EC is thought to impose structure on hippocampal firing sequences [8], to constrain generative replay to sequences that are probable and/or plausible. In other words, EC may define the low-dimensional embedding space (or manifold) through which hippocampal generative replay can traverse. By considering the sensory inputs received by EC, the function of EC can further be conceptualised as transforming egocentric sensory representations to allocentric latent variables represented by hippocampus [124–126].

This interaction between EC and hippocampus resembles that observed during theta (discussed above) where EC inputs to CA1 impose structure on theta sequences to ensure tractable latent state inference. This raises the intriguing possibility that structural codes in EC are iteratively learned from and applied to both theta sweeps and, in turn, generative replay during SWRs. Indeed, meaningful variation across both theta and SWR sequences may be learned by plasticity rules such as Spike-Time Dependent Plasticity or Behavioural Timescale Synaptic Plasticity [127]. For example, within a theta sweep future and past events are mapped onto pre- and postsynaptic activity respectively, within a time window concomitant with plasticity. During SWRs, where the time window for plasticity is further compressed, not only can plasticity occur between neighbouring events in a sequence but also between entirely disparate events that were spatiotemporally discontinuous in the waking period. Optogenetic perturbation of theta sequences during spatial navigation in a novel maze disrupts the sequential structure in SWRs [128], suggesting that theta sequences themselves may mediate plasticity required for subsequent replay (Figure 2a-b). To summarise, theta and SWR sequences therefore provide opportunity for plasticity within hippocampus which can provide a training signal to cortical circuits, to embed and refine a hierarchical generative model and in turn place constraints on generative sequences.

Comparisons between the biological brain and AI further emphasises the computational significance of generative replay. Notably, AI agents are often trained extensively using supervised learning algorithms that train an agent to predict labelled outputs. However, the brain rarely has access to explicit labels. Humans and animals are nevertheless able to rapidly and flexibly adapt to new environments. Applying self-supervised learning (SSL) to AI demonstrates that SSL can provide a means to efficiently explore real and hypothetical trajectories, to extract and learn an embedding space that captures meaningful factors of variation. Generative replay can provide the brain with a means to implement SSL, by replaying and traversing hypothetical trajectories, particularly after being exposed to a new experience.

## Perturbations to the hippocampal generative model in psychiatric disorders

Given the computational reach of hippocampal generative activity, failures of this system may account for an array of different symptoms reported in neuropsychiatric disorders, including symptoms that extend beyond memory impairment. In this final section, we use psychosis as a case study to explore how perturbations to the hippocampal generative model can account for core clinical symptoms, including hallucinations and delusions.

Psychosis is observed in several neuropsychiatric disorders and is considered a hallmark symptom of schizophrenia and bipolar disorder. Psychosis involves patients experiencing hallucinations and forming delusional beliefs. While behavioural assessments of these so called ‘positive symptoms’ are used for diagnosis, positive symptoms are typically also accompanied by both negative, and cognitive symptoms, including memory impairment [129]. Together these behavioural symptoms can be framed as impairments in perceptual and contextual inference, whereby individuals fail to appropriately segment experience into distinct latent causes [130–132]. In other words, patients have difficulty inferring *what* is causing *what* in the world. For example, delusions (e.g. ‘they are watching me’) may be attributed to under-segmenting latent causes, leading to failures in belief updating despite changes in the underlying latent cause. This results in inappropriate generalisation across contexts with poor separation between self and other, internal and external etc. Hallucinations (e.g. ‘I can hear a voice’) may similarly be attributed to inappropriate assignment of latent causes. Closely related constructs are probed in psychosis using behavioural paradigms such as reversal learning paradigms [133], volatility estimation, and belief updating under uncertainty [134]. However, here we highlight the need for more sensitive behavioural paradigms that explicitly aim to quantify disruption in latent state inference.

Framing behavioural symptoms in psychosis as an impairment in segmenting distinct latent causes provides some key predictions for our understanding of the underlying physiological mechanisms and has potential to guide development of novel treatment strategies. Current treatment for psychosis involves antipsychotic drugs that were largely discovered through serendipity. These drugs target excess dopamine via dopamine antagonists which focus predominantly on D2 receptors. While antipsychotic drugs can effectively reduce positive symptoms, such as hallucinations or delusions, treatment remains ineffective in around a third of patients and these drugs show no or limited effect on cognitive symptoms such as memory impairments [135]. This suggests that dopaminergic dysfunction alone is insufficient to account for the full spectrum of symptoms reported in psychosis. A broader framework, centered on the interaction between dopaminergic circuits and other brain regions, including the hippocampal generative model, may provide a more comprehensive account. One possibility is that a disordered interaction between hippocampus and dopaminergic circuits can account for impaired segmenting of distinct latent causes and perturbed updating of latent causes.

In individuals with psychosis, growing evidence suggests both structural and functional abnormalities in the hippocampus [136,137]. Structural changes in the hippocampus, such as volume decrease, are present in people at genetic risk for schizophrenia [138] and Genome Wide Association Studies (GWAS) show a genetic overlap between the risk for schizophrenia and lower hippocampal volume [139]. In addition to these structural changes, functional changes include hippocampal hyperactivity which is thought to be attributed to increased neural firing in excitatory pyramidal cells in the hippocampus [140–145]. This increased neural firing in excitatory pyramidal cells may in turn be attributed to pre- and postsynaptic abnormalities in GABAergic interneurons, particularly parvalbumin positive (PV+) GABAergic interneurons which show hypofunction in psychosis [146,147]. Additional GABAergic markers suggest abnormalities both in patients with psychosis and genetic risk carriers [145,148–150], indicating reduced cortical inhibitory firing and reduced hippocampal GABA concentrations. Abnormalities in hippocampal PV+ GABAergic inhibitory interneurons may reduce the selectivity of pyramidal cells, analogous to reduced selectivity of place fields observed in the developing hippocampus prior to PV+ interneuron maturation [151]. Across the population, reduced selectivity of place fields may manifest as disruption in remapping. Therefore, the hippocampus may fail to remap when new latent causes should be inferred, reflecting an impairment in segmenting latent causes.

This aberrant remapping due to reduced selectivity of hippocampal pyramidal neurons may be further exacerbated by the effect of hippocampal hyperactivity on downstream brain areas. For example, hippocampal hyperactivity may drive excess release of dopamine in dopaminergic ventral tegmental area (VTA) [152,153]. Excess release of dopamine may perturb prediction error signalling to hippocampus, further disrupting the mechanisms that support the formation of new latent states or transitions between existing states. Moreover, excess dopamine may assign inappropriate salience to inferred latent states in hippocampus, contributing to the persistence and strong conviction of hallucinations and delusional beliefs in psychosis.

Hippocampal hyperactivity may also account for changes in fine-tuned temporal sequences that support generative activity. Reduced theta power and reduced theta coherence are reported in both animal models of psychosis and people with lived experience of psychosis [154–158]. Gamma oscillations are often nested within theta cycles, forming theta-gamma phase-amplitude coupling that organizes hippocampal activity across time scales [159]. A reduction in the power of both gamma-band oscillations and theta-gamma phase amplitude coupling can be observed in psychosis, from people at clinical high risk to those with chronic schizophrenia [147,160–163]. In people at genetic risk for psychosis, reduced power of theta and gamma oscillation appears to result from a lack of age-expected maturation during adolescence, in agreement with a neurodevelopmental model of schizophrenia [164]. Consistent with a late maturation of PV+ interneurons [165], these changes in major oscillatory markers may be the direct consequence of PV+ hypofunction and associated hippocampal hyperactivity. Indeed, PV+ interneurons are reported to mediate phase coupling of spiking activity in hippocampal theta oscillations [166] and control temporal dynamics within gamma oscillations [167]. In psychosis, disturbances in hippocampal PV+ interneurons may therefore disrupt the precise timing of hippocampal theta sweeps and resulting predictions of upcoming sensory experience.

**Figure 3:**
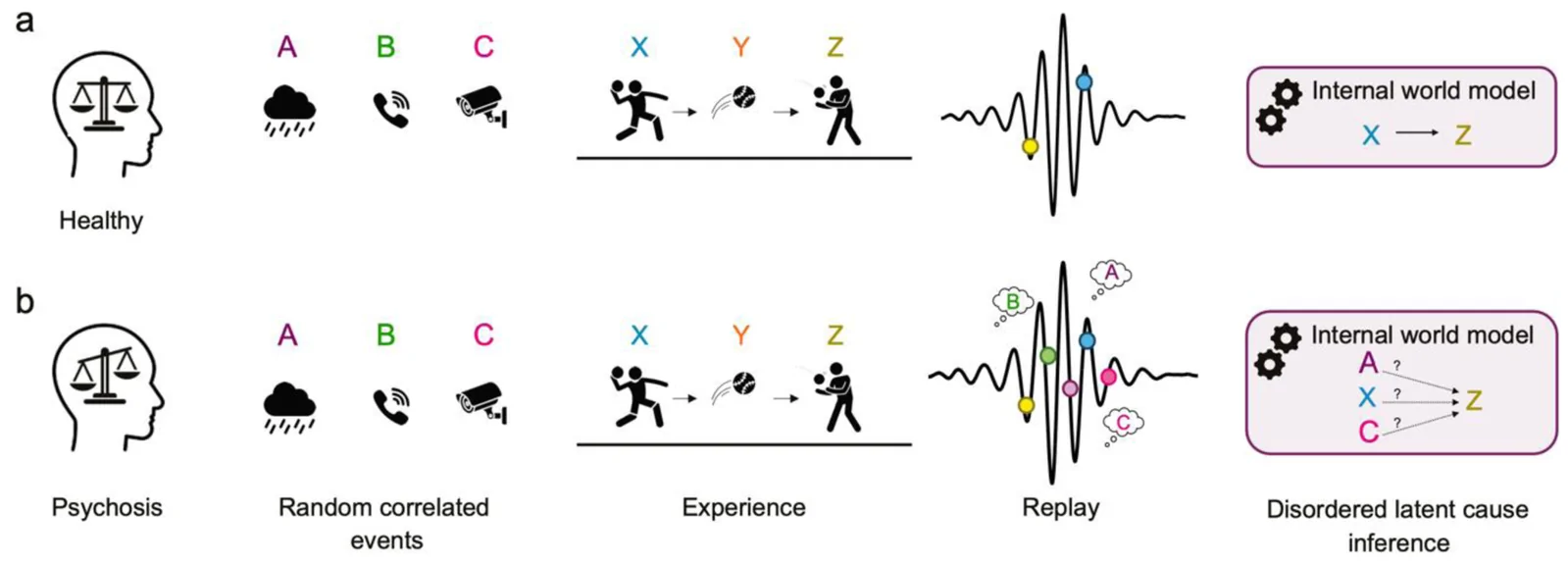
Perturbations in the hippocampal generative model explain core symptoms of psychosis **(a-b)** Schematics comparing hippocampal generative processing in healthy controls and psychosis. *X* corresponds to seeing a ball being thrown, *Y* to tracking the ball’s trajectory, and *Z* to seeing the ball get caught. *A, B, C* are random correlated events: *A* is rain, *B* is a phone call in the background, and *C* is a security camera in the vicinity. **(a)** Schematic for healthy controls. Random correlated events are not replayed in SWRs during periods of rest or sleep. Latent causes are inferred correctly (p(Z|X)). **(b)** Schematic for people with psychosis. Due to reduced effect of inhibitory interneurons, hippocampal hyperactivity allows random correlated events to be coactivated during SWR replay, leading to spurious sequences, disordered latent cause inference (p(Z|A) or p(Z|X) or p(Z|C)) and the formation of false beliefs. In this example they might infer that the ball is caught (Z) because someone is watching through the security camera (C) and not because it was thrown (X).

A similar logic can be applied to SWR firing sequences where gross changes in SWR power are observed in psychosis. For example, in mouse models of schizophrenia the rate of SWRs is elevated [168,169], while replay in patients with psychosis is less faithful to direct experience [170,171]. These reports suggest that hippocampal hyperactivity promotes spurious firing patterns that allow for aberrant or “over-extended” sequences. These aberrant sequences may account for core symptoms in psychosis (Figure 3). For example, the interaction between aberrant sequences and excess dopamine may provide a mechanism to misattribute salience or value to previously neutral stimuli [172,173]. The interaction between hippocampal hyperactivity and dopaminergic circuits during SWRs may therefore provide a mechanism to ***misattribute credit or value*** in a manner that produces highly salient and rigid spurious latent causes. Taken together, failures in the physiological implementation of the hippocampal generative model may account for core symptoms in psychosis, providing testable prediction for the mechanistic basis of the disorder.

## Manipulating the hippocampal generative model

Recent advances in neuroscience and non-invasive brain stimulation now allow for causal manipulation of activity in the hippocampus, providing opportunity to experimentally manipulate generative activity. As discussed above, TMR provides a well-established method to selectively bias the content of replay during periods of rest and sleep. Beyond sensory cueing, emerging non-invasive brain stimulation techniques permit direct perturbation of hippocampal dynamics. Transcranial focused ultrasound stimulation (TUS) can noninvasively target deep-brain structures, including the hippocampus, with unprecedented spatial precision [174]. Preclinical studies indicate that TUS can modulate hippocampal oscillatory coupling, including theta–gamma phase–amplitude interactions, to alter behavioural measures of both encoding and memory retrieval [175]. In humans low-intensity, focal TUS can causally modulate hippocampal activity and selectively disrupt participants ability to perform inference [176], establishing a powerful approach to further probe how the human hippocampus contributes to a generative model. Similarly, temporal interference stimulation (TI) can selectively modulate activity in deep structures, such as hippocampus, by generating interference envelopes at behaviourally relevant frequencies (e.g., theta or ripple frequency range) through the superposition of multiple high-frequency fields [177]. Experimental work demonstrates that temporally patterned interference stimulation can entrain hippocampal oscillations and modulate memory-guided behaviour [178,179] providing a means to modulate generative replay. Together, these approaches offer promising opportunities to further characterize the hippocampus as a generative model and establish tools to manipulate the hippocampal generative model in patient groups.

## Concluding Remarks

Conceptualizing the hippocampus as a generative model offers a unifying framework for understanding the multifaceted role of the hippocampus in cognition. Beyond serving memory or representing a cognitive map, we propose that the hippocampus engages in perceptual inference by representing latent causes for sensory experience. Using these latent causes, the hippocampus has the capacity to then generate sequences that simulate possible futures, during both theta sequences and SWRs. This dual capacity to perform perceptual inference and generate new data samples enables the hippocampus to support a wide range of cognitive functions, including memory, prediction, imagination, planning, inferential choice, and generalization. We further explore how perturbations to these generative processes may account for core symptoms reported in psychosis where internally generated experiences are mistaken for reality. Viewing the hippocampus as a generative model may therefore explain how the hippocampus contributes to higher-order cognition while also offering a powerful framework to identify physiological mechanisms that underlie behavioural symptoms in psychiatric disorders.

**Predictions**

**1: Hippocampal theta sweeps reflect generative sampling**

Hippocampal spiking activity in late theta phases reflects generative sampling of future inferred latent states. This ‘online’ generative activity can rapidly predict upcoming behaviour, over a short-time horizon. Silencing CA3 in late theta phases will impair behaviours that rely on short-term planning, as recurrent connectivity in CA3 endows CA1 sequences with online generative capacity.

**2: Internally generated prediction errors can be used to update the generative model during offline periods of rest/sleep:**

Internally generated prediction error signals are computed in the hippocampus during rest/sleep, in the absence of sensory input. These prediction error signals are generated using the mismatch between: ***(a)*** hippocampal latent state representations used to simulate sensory data in low levels of the cortical hierarchy; and ***(b)*** *inferred* hippocampal latent state representations, where the simulated sensory data provides input that can be used for perceptual inference. These prediction error signals can be used to update neural circuits, including the parameters of the generative model itself.

**3: Dopamine projections to hippocampus are necessary to infer new latent states in the hippocampus**

A sufficiently large dopaminergic prediction error signal can drive formation of a new latent state representation in the hippocampus. Blocking dopaminergic projections to hippocampus (deriving from VTA and/or LC) will impair inference of new latent states. For example: in rapid extinction paradigms, this manipulation will reduce expression of renewal behaviour; following changes in context, the probability of global remapping will be reduced; on a new task, splitter cells may not emerge to represent hidden latent states.

**4: Generative replay plays a causal role in assigning credit to previously neutral and/or non-contiguous stimuli**

Generative replay sequences that start with representation of reward provide opportunity to assign credit to previously neutral or non-contiguous cues by ‘back-propagating’ value to other cues. Dopaminergic projections to hippocampus are necessary for this mechanism. Therefore, blocking dopaminergic projections to hippocampus will impair credit assignment to neutral or non-contiguous cues, while sparing learning for directly reinforced associations.

**5: Structural codes in mEC are iteratively learned from and applied to theta sweeps, and in turn constrain the structure of generative replay during SWRs**

Low-dimensional representations in mEC can impose structure on hippocampal activity. These structural codes are iteratively learned from and applied to hippocampal spiking sequences in theta, during active behaviour. In turn, mEC may constrain the structure of generative replay during offline SWRs. Silencing mEC during hippocampal SWRs will release this constraint, allowing hippocampal sequences to deviate from plausible and optimized future narratives.

**6: Symptoms reported in psychosis can be explained by impaired segmentation of latent states**

In psychosis, mechanisms that contribute to the formation of delusional beliefs include: ***(a)*** hyperactivity in the hippocampus, leading to aberrant or “over-extended” generative activity (in both theta and SWRs) that has potential to bind non-contiguous stimuli and/or misattribute credit; ***(b)*** impaired dopaminergic signalling, which under-segments latent state representations in hippocampus, and attributes excess salience to inferred latent states, accounting for persistent and strong convictions of delusional beliefs.

## Data Availability

No data or code were used in generating this manuscript.
